# Live-attenuated PruΔ*gra72* strain of *Toxoplasma gondii* induces strong protective immunity against acute and chronic toxoplasmosis in mice

**DOI:** 10.1186/s13071-024-06461-9

**Published:** 2024-09-05

**Authors:** Jing Li, Yu Kang, Ze-Xuan Wu, Shu-Feng Yang, Yu-Yang Tian, Xing-Quan Zhu, Xiao-Nan Zheng

**Affiliations:** Laboratory of Parasitic Diseases, College of Veterinary Medicine, Shanxi Agricultural University, Taigu, Jinzhong, Shanxi Province 030801 People’s Republic of China

**Keywords:** *Toxoplasma gondii*, PruΔ*gra72*, Live-attenuated vaccine, Acute and chronic infection, Immune responses

## Abstract

**Background:**

*Toxoplasma gondii* is an intracellular opportunistic pathogenic protozoan that poses serious threats, particularly in immunocompromised individuals. In the absence of a robust prophylactic measure, the mitigation and management of toxoplasmosis present formidable challenges to public health. We recently found that GRA72 plays an important role in parasitophorous vacuole (PV) morphology, growth and virulence of *T. gondii*. However, whether *gra72*-deficient strain can be used as a vaccine remains unknown.

**Methods:**

We first examined the attenuated virulence of *gra72* gene knockout strain (PruΔ*gra72*) and the parasite load in organs of the infected mice. Subsequently, we evaluated the immune-protective effects of the PruΔ*gra72* vaccination against challenge with various types of *T. gondii* tachyzoites and Pru cysts. Furthermore, levels of antibodies and cytokines induced by PruΔ*gra72* vaccination were examined. Statistical analysis was conducted by Student’s t-test or Mantel-Cox log-rank test based on data obtained from three independent experiments with GraphPad Prism 8.0.

**Results:**

We found that PruΔ*gra72* strain exhibited a significantly attenuated virulence even at the highest dose of 5 × 10^7^ tachyzoites in Kunming mice model. The significant decrease of brain cyst burden and parasite load in the organs of the PruΔ*gra72*-infected mice suggested its potentiality as a live-attenuated vaccine. Hence, we explored the protective immunity of PruΔ*gra72* vaccination against toxoplasmosis. Results showed that vaccination with 5 × 10^6^ PruΔ*gra72* tachyzoites triggered a strong and sustained Th1-biased immune response, marked by significantly increased levels of anti-*T. gondii* IgG antibodies, and significantly higher levels of Th1 type cytokines (IL-2, IL-12 and IFN-γ) compared to that of Th2 type (IL-4 and IL-10). Vaccination with 5 × 10^6^ PruΔ*gra72* tachyzoites in mice conferred long-term protection against *T. gondii* infection by less virulent tachyzoites (ToxoDB#9 PYS and Pru strains) and Pru cysts, provided partial protection against acute infection by high virulent Type I RH tachyzoites and significantly decreased brain cyst burden of chronically infected mice.

**Conclusions:**

The avirulent PruΔ*gra72* induced strong protective immunity against acute and chronic *T. gondii* infection and is a promising candidate for developing a safe and effective live-attenuated vaccine against *T. gondii* infection.

**Graphical Abstract:**

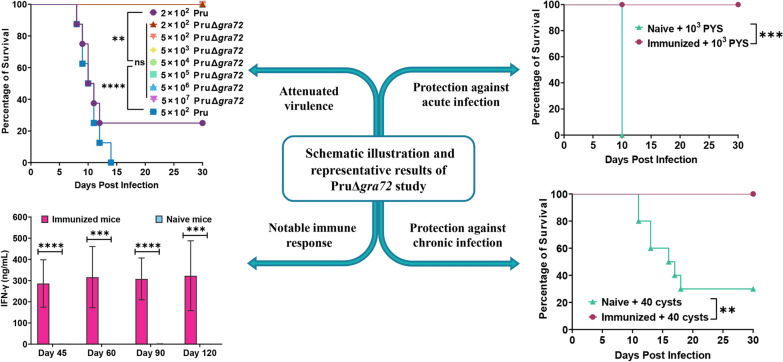

**Supplementary Information:**

The online version contains supplementary material available at 10.1186/s13071-024-06461-9.

## Background

Toxoplasmosis, a prevalent zoonotic parasitic disease, emanates from *Toxoplasma gondii* and endangers practically all warm-blooded animals, including humans [1]. Humans and animals are predominantly infected with *T. gondii* by ingesting tissue cysts or oocysts from feline excrement or through congenital routes infiltrating via the placental barrier [2]. Immunocompetent individuals typically remain asymptomatic, yet infections with virulent *T. gondii* strains in immunocompromised hosts may result in severe clinical manifestations, such as encephalitis, pneumonia, retinitis and myocarditis, particularly in pregnant women susceptible to fetal deformities or miscarriage [2, 3]. Currently, *T. gondii* remains a major hindrance to global livestock development and public health. *Toxoplasma gondii* is the only recognized species within the genus *Toxoplasma* with approximately 300 different genotypes, including the three major clonal lineages (Types I, II and III) which have distinct virulence in mice [4–6].

The effective control and prevention of zoonotic toxoplasmosis present significant challenges, given the intricate multi-stage lifecycle of *T. gondii*, the diversity of intermediate hosts and its potent evasion of the host immune system [7, 8]. Though combined therapy of pyrimethamine and sulfadiazine has been proven to be the most effective in treating tachyzoites, pharmacological interventions often entail various adverse effects and fail to completely eradicate bradyzoites, thereby facilitating latent infections and drug residues [9]. The development of effective vaccines has emerged as a primary strategy for preventing and managing this disease. Thus far, numerous *T. gondii* vaccines have been devised, including nucleic acid vaccines, protein vaccines, nanovaccines, live vector-based vaccines and live attenuated vaccines [8]. Among these, live-attenuated vaccines, which mimic the entire process of parasite infection in the host, stimulate the host’s immune system to provide effective immune protection [8, 10]. *Toxoplasma gondii* live vaccines, including strains S48, T-263 and TS-4, have demonstrated efficacy in bolstering host immunity against *T. gondii* infection [11]. Notably, the T-263 strain elicits robust immune responses in feline hosts, especially showing commendable efficacy in preventing oocyst shedding [12, 13]. Meanwhile, vaccination with the S48 strain serves as a prophylactic measure against congenital toxoplasmosis in ovine populations, concurrently mitigating parasitic burden in meat products and reinforcing overall food safety [11]. However, the S48 strain carries the risk of mutations and restoration of pathogenicity [8]. Presently, Toxovax®, derived from the S48 strain lineage, stands as the only commercially endorsed vaccine available in countries like New Zealand and the UK [14]. Despite considerable progress achieved in the aforementioned vaccine types, live-attenuated *T. gondii* vaccines have not yet achieved complete elimination of tissue cysts and eradication of toxoplasmosis [15]. Further research into efficient and deployable vaccines remains an urgent matter to be addressed.

With the extensive development and application of genetic engineering techniques, live-attenuated *T. gondii* mutants were recently constructed by deleting key genes associated with virulence or metabolism, and their protective efficacy against *T. gondii* was explored. The RHΔ*tkl1* strain elicits robust humoral and cellular immune responses in immunocompetent mice, effectively counteracting *T. gondii* infection [16]. Inoculation with ME49Δ*cdpk3* significantly attenuates *in vivo* virulence in murine hosts, markedly diminishes cyst formation and provides a pivotal protection against both acute and chronic *T. gondii* infections by diverse strains [17]. Additionally, the double-gene knockout strain lacking *gra17* and the newly putative transporter gene (*npt1*) confers protection against *T. gondii* infection in mice by modulating inflammatory responses [18].

Dense granule proteins (GRAs), indispensable secretory proteins of *T. gondii*, are involved in regulating important physiological activities, such as host cell immune defense, protein transport, evasion and chronic infection [19]. Secreted upon host invasion, GRAs dissolve within the parasitophorous vacuole (PV) lumen, localize to the intravacuolar network (IVN) embedded the PV membrane, insert into the parasitophorous vacuole membrane (PVM) or are exported into the host cell to exert their effects [19]. GRA42 and GRA43 serve as protein partners, mediating the correct localization of various proteins such as GRA17, GRA23 and GRA35 to the PVM [20]. GRA39 is involved in lipid utilization, and the absence of GRA39 reduces the virulence of *T. gondii* in the host, resulting in a lower cyst burden [21]. GRA24 possesses the ability to prolong the autophosphorylation of p38α and can activate the expression of pro-inflammatory genes in macrophages [22]. These identified GRAs have been validated as promising candidates for the development of vaccines against *T. gondii* [23–25].

Recently, our study showed that GRA72, possibly interacting with GRA47, is important for PV morphology, growth and virulence of *T. gondii* [26], consistent with a recent research [27]. Parasites deficient in GRA72 form abnormal morphology (bubble) PV, akin to Δ*gra17* parasites [28]. Additionally, GRA72 is also indispensable for the growth and virulence of *T. gondii* [28] and participates in the proper trafficking of GRA17 and GRA23 on the PVM [29]. Deletion of *gra72* gene in Pru strain attenuated the virulence to mice at an infective dose of 1 × 10^6^ tachyzoites [26]. Nevertheless, the potential of PruΔ*gra72* serving as a candidate vaccine for preventing toxoplasmosis remains unknown. Thus, the present study explored the immune-protective potential of the PruΔ*gra72* strain and evaluated the roles of humoral and cellular immunity in its protection against acute and chronic *T. gondii* infection in mice.

## Methods

### Mice and parasites

Eight-week-old female Kunming mice, susceptible to acute and chronic *T. gondii* infection compared to BALB/c and C57BL/6 mice [30], were purchased from Beijing Sibeifu Biotechnology Co., Ltd. All mice were fed under specific pathogen-free conditions at 50–60% humidity and 25 °C, provided with adequate food and water ad libitum. Mice were acclimatized for 1 week prior to the commencement of the experimental studies. Animal experiments were conducted following the principles of minimizing animal suffering and protecting animal welfare as much as possible. The tachyzoites of *T. gondii* Type I RH strain, Type II Pru strain, ToxoDB#9 PYS strain, the parental PruΔ*ku80* strain (referred to as Pru) and the constructed gene knockout PruΔ*ku80*Δ*gra72* strain (referred to as PruΔ*gra72*) [26] were replicated in human foreskin fibroblast (HFF) cells, maintained in DMEM containing 2% fetal bovine serum (FBS) and cultured in a CO_2_ incubator at 37 °C with 5% CO_2_. The cysts of the Type II Pru strain were obtained from the brain homogenate of infected mice, as described previously [31].

### Optimization of PruΔ*gra72* vaccination dose

To assess the virulence of the PruΔ*gra72* strain *in vivo*, 8-week-old Kunming mice were infected by intraperitoneal injection with different doses (2 × 10^2^, 5 × 10^2^, 5 × 10^3^, 5 × 10^4^, 5 × 10^5^, 5 × 10^6^ and 5 × 10^7^) of PruΔ*gra72* tachyzoites or (2 × 10^2^ and 5 × 10^2^) of wild-type Pru tachyzoites (8 mice per group). The clinical toxoplasmosis symptoms and mortality of all infected mice were observed twice every day for 30 days post-infection (dpi). The number of brain cysts in mice surviving at 30 days were counted. The presence of *T. gondii* in mice brain was determined with PCR targeting *T. gondii B1* gene as previously described [32].

The parasite loads in organs (including eyes, brain, heart, liver, spleen, lungs, kidneys and intestines) of mice infected by 5 × 10^6^ PruΔ*gra72* or Pru tachyzoites were determined 7 days post-infection using quantitative polymerase chain reaction (qPCR). Tissue DNA of various organs of infected mice was extracted using the TIANamp Genomic DNA Kit (Tiangen Biotech, DP304-03, Beijing, China). Sample DNA underwent normalization utilizing the mice U6 gene using ChamQ Universal SYBR qPCR Master Mix (Vazyme Biotech, Q711-02, Nanjing, China) to derive the CT values. Subsequently, parasite load was calculated using the standard curve analysis based on the Lg (tachyzoite number) and the corresponding CT values of the 529-bp fragment of extracted DNA from different gradient tachyzoites.

### Protection against acute and chronic infection

Mice were intraperitoneally vaccinated with 5 × 10^6^ PruΔ*gra72* tachyzoites or mock vaccinated with the same volume of PBS. To investigate the protective effect of PruΔ*gra72* immunization against acute *T. gondii* infection in mice, 10^2^ and 10^3^ RH or PYS tachyzoites along with 5 × 10^4^ Pru tachyzoites (6 mice per group) were intraperitoneally injected into immunized and control mice at 60 and 120 days post-vaccination (dpv). The viability and number of tachyzoites injected into mice were examined by using the *in vitro* plaque assay [18].

For protection assessment against chronic infections, mice were inoculated with either 10 or 40 cysts (10 mice per group) at 60 and 120 dpv. The mortality and clinical toxoplasmosis symptoms were recorded within 1 month. At 30 dpi, the mouse cysts obtained from chronically infected mice were calculated under microscopic examination. Brain tissues without cysts were examined for *B1* gene to further determine the infection status in mice.

### Preparation of soluble *T. gondii* antigen (STAg)

The freshly harvested tachyzoites were washed three times with pre-cold PBS and resuspended in an appropriate volume of PBS, and the cellular integrity was disrupted by multiple freeze-thaw cycles. The suspension was sonicated on ice at a power of 80 W/s for 30 min to ensure efficient disruption and release of intracellular components. Following sonication, the suspension was centrifuged at 12,000 × *g* for 10 min to collect the supernatant containing the STAg and stored at -80 °C to maintain its stability and integrity.

### Evaluation of *T. gondii*-specific antibodies in PruΔ*gra72*-immunized mice

Immunological evaluations were carried out at 45, 60, 90 and 120 dpv to reveal differences in humoral immune responses between the vaccinated and unvaccinated groups. Total IgG and subclasses of IgG were detected using the mouse serum samples by ELISA to indicate humoral immune response profiles as previously described [33]. Briefly, 100 μl STAg per well, diluted to 1 μg/100 μl, was added to wells of a 96-well plate, followed by incubation at 37 °C for 2 h, then coated overnight at 4 °C. The antigen-coated ELISA plate was washed three times with 0.5% PBST, and the final wash was pat-dried before proceeding. Subsequently, non-specific binding sites were blocked by incubating with 5% BSA in a 37 °C incubator for 1 h, followed by another round of washing. Serum samples, diluted 1:100 in 1% BSA, were added to the wells and incubated at 37 °C. Next, the HRP-conjugated goat anti-mouse IgG (Abcam, AB97040, Cambridge, UK) was diluted at a ratio of 1:3000, while the goat anti-mouse IgG1 (Abcam, AB98693, UK) and IgG2a (Abcam, AB98698, UK) were diluted at a ratio of 1:5000 and then added to each respective well. After washing three times, TMB (3, 3′, 5, 5′-tetramethylbenzidine) Chromogen Solution for ELISA (Beyotime Biotech, P0209-100 ml, Shanghai, China) was used for color development. Upon stabilization of color development, a 2% sulfuric acid solution was added to terminate the reaction, and the optical density (OD) was measured at 450 nm.

### Detection of cytokines in splenocyte supernatants in PruΔ*gra72*-immunized mice

Following a previously described study [33], the spleens of immunized and non-immunized mice were gathered using sterile surgical forceps to prepare splenocyte suspensions for cytokine determination to discern cellular immune responses. The retrieved spleen was washed with RPMI-1640, gently ground on a 200-mesh nylon mesh to obtain a cell suspension and centrifuged at 1500 × *g* for 10 min to precipitate cellular debris. All separated splenic cells were immersed in red blood cell lysate for 3 min to procure a homogeneous splenic cell suspension. The suspension was then resuspended in RPMI-1640 culture medium supplemented with 10% FBS. The live cell number was determined utilizing the trypan blue exclusion method to ensure > 95% cell viability for subsequent analysis. The concentration of splenic lymphocytes was adjusted to 3 × 10^6^ cells/ml and dispensed into individual wells. The splenocytes were then stimulated with STAg at a final concentration of 10 μg/ml to elicit an immune response. Following stimulation, the supernatants were harvested at specific time intervals: 24 h for IL-2 (BioLegend, 431,007, San Diego, USA) and IL-4 (BioLegend, 431,107, USA) assessment, 72 h for IL-10 (BioLegend, 431,417, USA) assessment and 96 h for IL-12 (BioLegend, 433,607, USA) and IFN-γ quantification (BioLegend, 430,807, USA). The collected supernatant was used to test the cytokine levels following the recommendations of the above-mentioned kits.

### Statistical analysis

All experimental data were obtained for three biological replicates and analyzed using GraphPad Prism 8.0 (GraphPad Software Inc., La Jolla, CA, USA). A two-tailed, unpaired Student’s t-test was employed to determine the significance of differences between two groups, including antibody levels, cytokine levels, cyst burden and parasite burden. The Mantel-Cox log-rank test is applied to assess differences in survival curves. Here, *P* < 0.05 was considered statistically significant, and *P* < 0.01, < 0.001, < 0.0001 represented varying degrees of significance.

## Results

### Attenuated virulence and optimization of vaccination dose in mice

GRA72 plays important roles in PVM permeability and growth of *T. gondii* [26, 28]. Knockout of *gra72* in Type II strain attenuated the parasite virulence [26, 28]. To determine whether this live-attenuated strain confers protection against *T. gondii*, the gene knockout PruΔ*gra72* strain constructed in our previous study was used in this study. The PCRs and the enlarged bubbled PVs of PruΔ*gra72* used in this study were consistent with that of PruΔ*gra72* in our previous study [26] (Additional file 1: Figure S1).

**Fig. 1:**
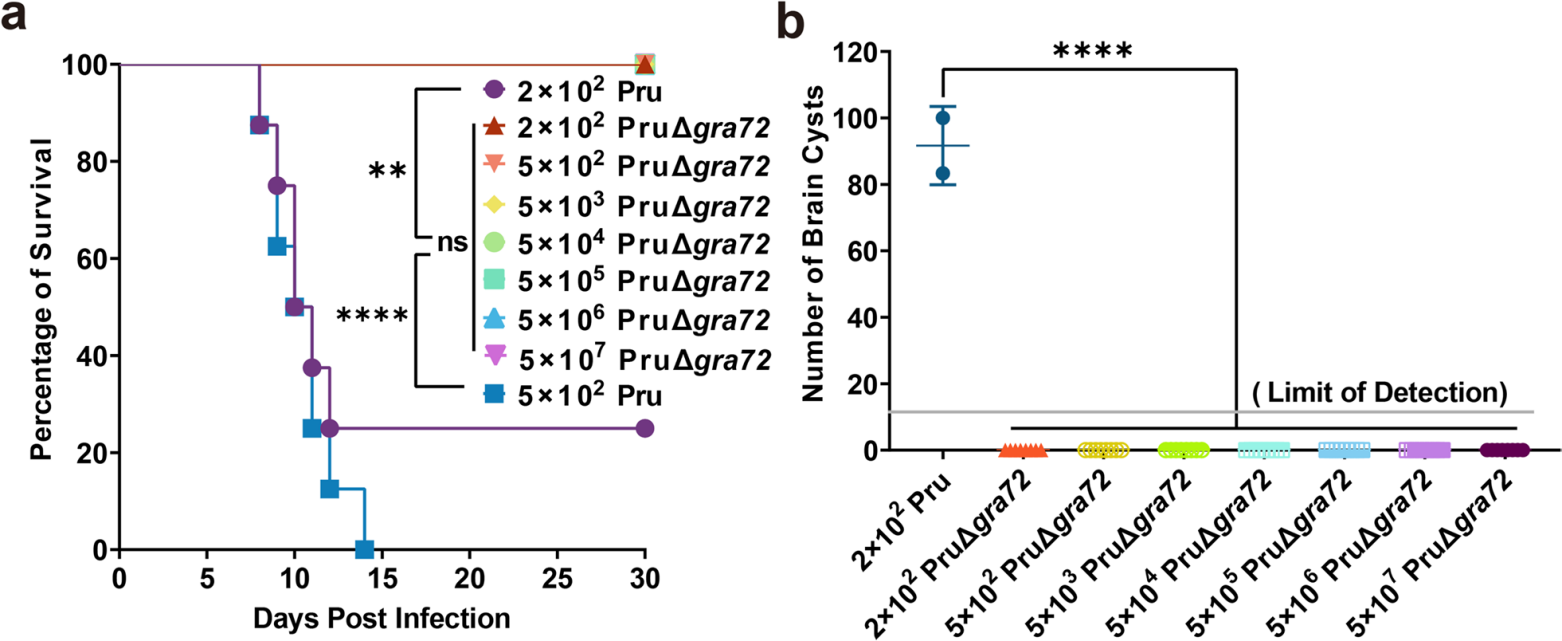
Virulence assessment of PruΔ*gra72* knockout strain in mice. (**a)** Survival curves of Kunming mice intraperitoneally infected with escalating doses (2 × 10^2^, 5 × 10^2^, 5 × 10^3^, 5 × 10^4^, 5 × 10^5^, 5 × 10^6^ and 5 × 10^7^) of PruΔ*gra72* or (2 × 10^2^ and 5 × 10^2^) wild-type Pru tachyzoites. ****P < 0.0001, **P < 0.01. (**b)** Cyst numbers in brain tissues detected in the infected mice survived at 30 days post-infection. ****P < 0.0001

To assess the virulence and the potential of PruΔ*gra72* as a live-attenuated vaccine, varying dosages of PruΔ*gra72* tachyzoites were intraperitoneally administered in Kunming mice. Mice injected with the PruΔ*gra72* strain maintained a 100% survival rate even at the highest dose of 5 × 10^7^ PruΔ*gra72* tachyzoites. However, all mice infected with 5 × 10^2^ Pru tachyzoites were killed, and those with 2 × 10^2^ Pru tachyzoites exhibited only a 25% survival rate (Fig. 1a). With the exception of mild messy fur observed in mice infected with the highest dose of 5 × 10^7^ gene knockout tachyzoites, PruΔ*gra72*-injected mice displayed no discernible clinical manifestations, while the Pru-infected mice manifested severe illness, such as lethargy, muscle weakness and decreased appetite within a week, ultimately leading to mortality. The surviving mice inoculated with 2 × 10^2^ tachyzoites of the wild-type strain displayed an average cyst burden of 92 ± 12, whereas no cysts were detected in the brains of mice infected with 5 × 10^7^ PruΔ*gra72*. Cyst burden significantly decreased in mice inoculated with the PruΔ*gra72* strain compared to that of the Pru-infected group (*P* < 0.0001) (Fig. 1b). To reveal whether the PruΔ*gra72* parasites reach the brains of the infected mice, the brains of all surviving mice infected with PruΔ*gra72* were examined by amplifying the *T. gondii B1* gene using PCR. The results showed that the positive rates of *B1* gene in the brains of these mice with infection doses of 2 × 10^2^, 5 × 10^2^, 5 × 10^3^, 5 × 10^4^, 5 × 10^5^, 5 × 10^6^ and 5 × 10^7^ were 12.5%, 12.5%, 25.0%, 25.0%, 62.5%, 87.5% and 87.5%, respectively (Additional file 2: Figure S2 and Additional file 3: Table S1). The positive rates of brain *B1* gene were positively correlated with the infection doses. These results indicate that although the PruΔ*gra72* parasites could reach the mouse brain, they fail to form cysts. Collectively, these results revealed a significant attenuation of the virulence of PruΔ*gra72* to mice.

**Fig. 2:**
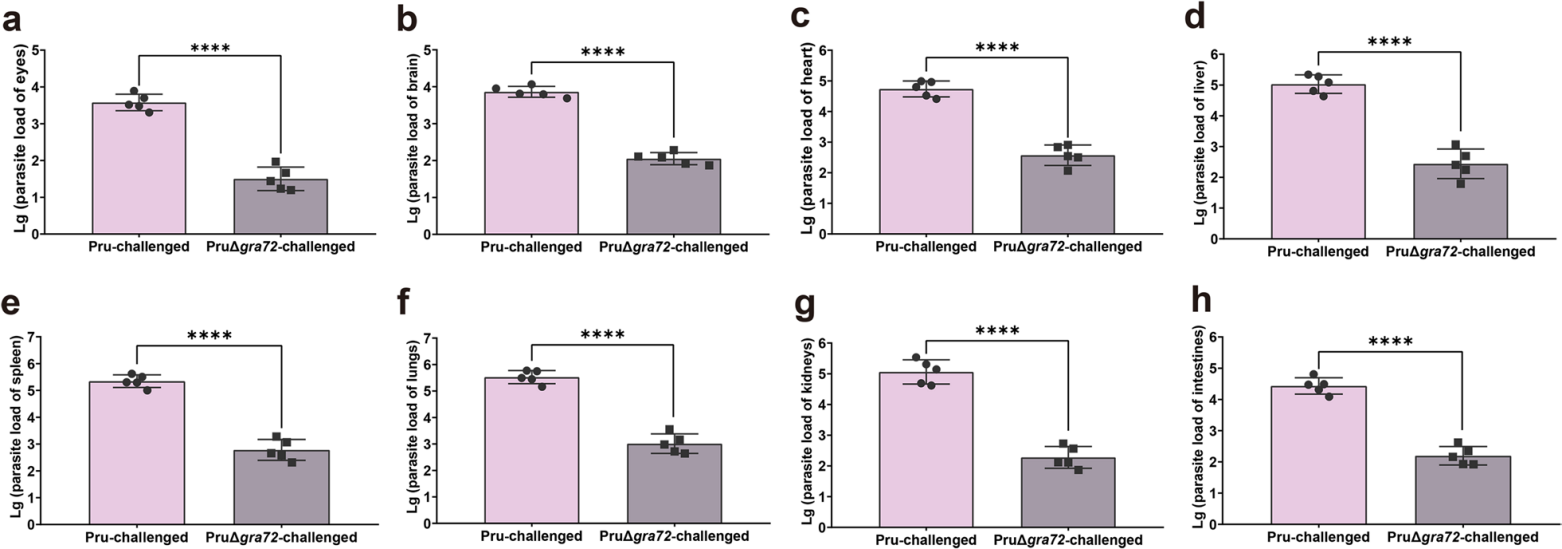
Reduced parasite burden in various murine organs of mice infected with PruΔ*gra72* tachyzoites. Parasite burden in the eyes (**a)**, brain (**b)**, heart (**c)**, liver (**d)**, spleen (**e)**, lungs (**f)**, kidneys (**g)** and intestines (**h)** of the mice inoculated with 5 × 10^6^ Pru or PruΔ*gra72* tachyzoites at 7 days post-infection, which were assessed by quantitative polymerase chain reaction (qPCR) (*n* = 5 mice per group). ****P < 0.0001

The parasite loads in various organs of the infected mice challenged with 5 × 10^6^ PruΔ*gra72* or Pru tachyzoites at 7 dpi, including eyes, brain, heart, liver, spleen, lungs, kidneys and intestines, were obtained. PruΔ*gra72*-infected mice exhibited parasite loads ranging from 10^1^ to 10^3^, while those infected with the wild-type Pru tachyzoites displayed parasite loads ranging from 10^3^ to 10^5^ (Fig. 2), showing the marked reduction of the parasite proliferation within the different organs in PruΔ*gra72*-infected mice (Fig. 2). These results indicate that the absence of the *gra72* gene has a profound impact on the *in vivo* replication and virulence of the parasite, and PruΔ*gra72* has potential to be used as a live-attenuated vaccine. Based on previous studies of the live attenuated RHΔ*ompdc*Δ*uprt* and PruΔ*gra17* strains [34, 35], we chose the immunization dose of 5 × 10^6^ PruΔ*gra72* tachyzoites for further research.

### PruΔ*gra72* confers protection against acute infection with various genotypes of *T. gondii* tachyzoites in mice

**Fig. 3:**
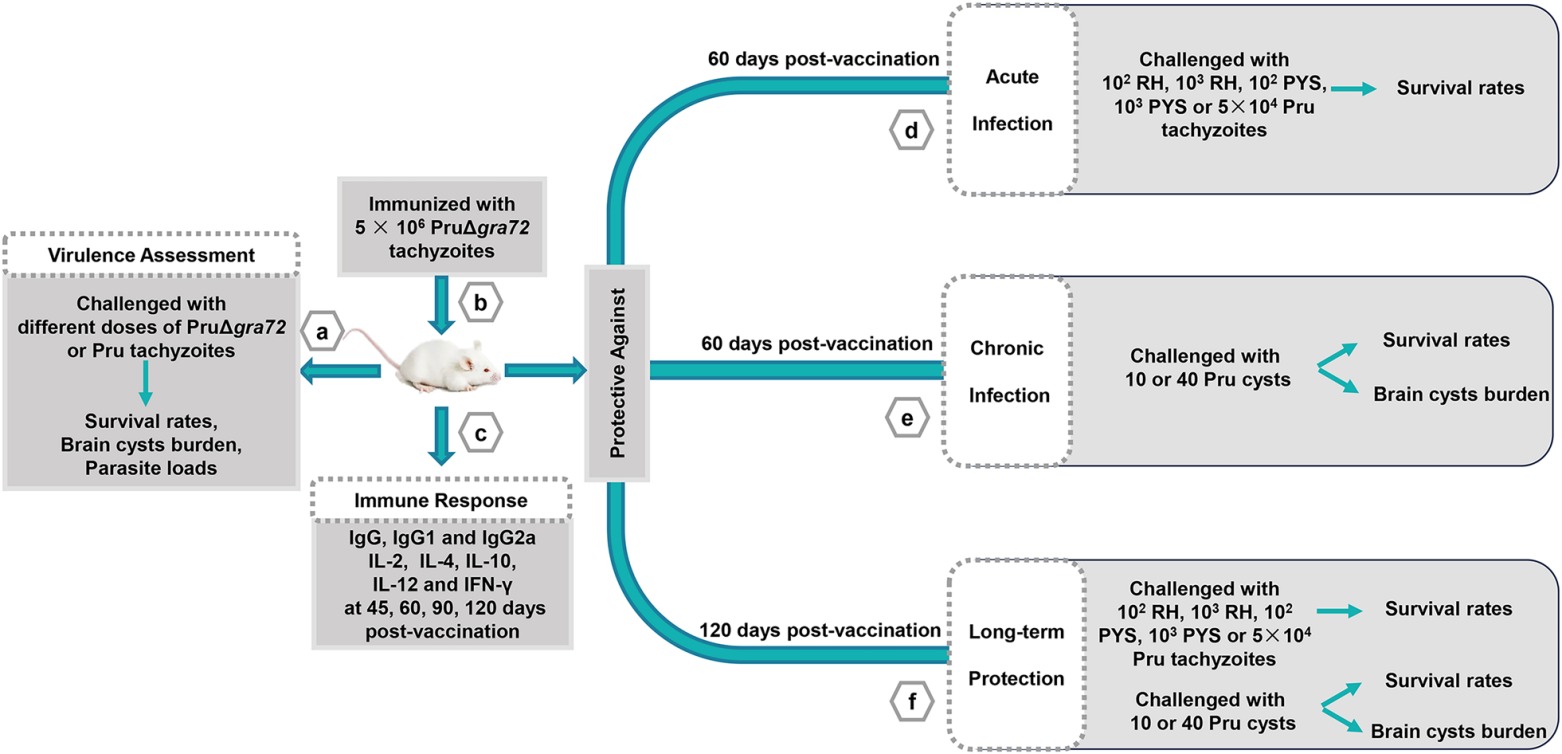
Schematic illustration of the study design assessing the potential of PruΔ*gra72* as a live-attenuated vaccine. Virulence assessment of PruΔ*gra72* (**a**), vaccination with PruΔ*gra72* tachyzoites (**b**), immune responses induced by vaccination (**c**), protection assessment against acute infection by *Toxoplasma gondii* tachyzoits (**d**), chronic infection by Pru cysts (**e**) and long-term *T. gondii* infection (**f**)

**Fig. 4:**
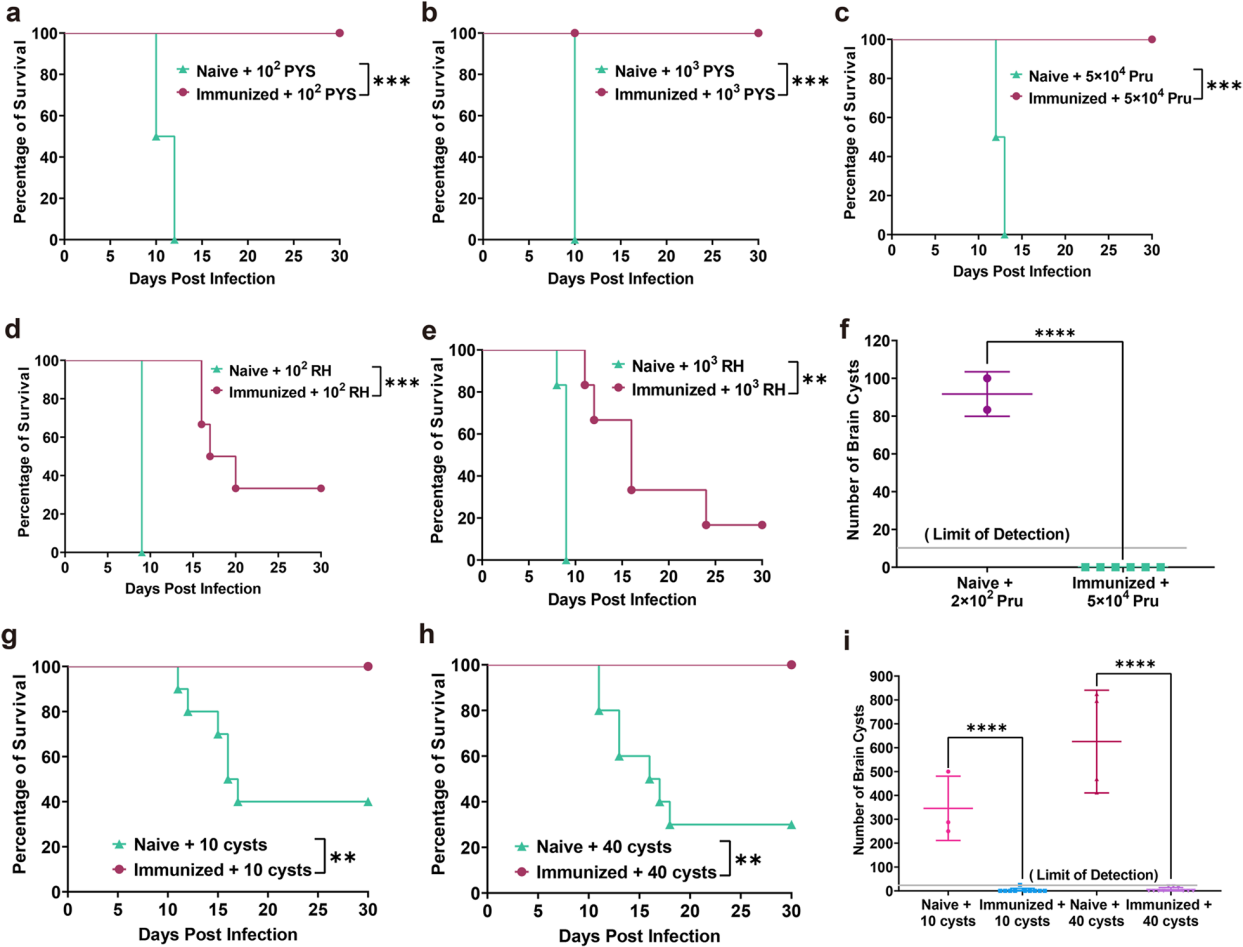
Immunization with PruΔ*gra72*-induced protection against acute and chronic *Toxoplasma gondii* infection at 60 days post-vaccination (dpv). Mice immunized with 5 × 10^6^ PruΔ*gra72* tachyzoites were challenged with varying doses of *T. gondii* tachyzoites, including 10^2^ PYS tachyzoites (**a**), 10^3^ PYS tachyzoites (**b**), 5 × 10^4^ Pru tachyzoites (**c**), 10^2^ RH tachyzoites (**d**) and 10^3^ RH tachyzoites (**e**). Survival rates were monitored for 30 days (*n* = 6 mice per group). ***P < 0.001, **P < 0.01. (**f**) Brain cyst numbers of the surviving mice infected with 5 × 10^4^ Pru tachyzoites at 30 days after infection. ****P < 0.0001. Additionally, mice were orally infected with 10 or 40 Pru cysts (**g**–**h**), and their survival rates were observed over 1 month (*n* = 10 mice per group) **P < 0.01. **i** Brain cyst burden of the surviving mice challenged with cysts at 30 days after infection. ****P < 0.0001

To determine the protective efficacy of PruΔ*gra72* vaccination against *T. gondii* acute infection (caused by tachyzoites), naive and vaccinated Kunming mice were challenged with 5 × 10^4^ Type II (Pru strain) tachyzoites, 10^2^ or 10^3^ ToxoDB#9 (PYS strain) tachyzoites and 10^2^ or 10^3^ Type I (RH strain) tachyzoites at 60 dpv (Fig. 3). The results showed that the immunized mice achieved 100% survival when challenged with different doses of PYS and Pru tachyzoites compared to the blank mice, which were all killed within 13 days (Fig. 4a–c). By challenging mice with RH tachyzoites, PruΔ*gra72* extended mouse survival time, with survival rates ranging from 16.7% to 33.3% (Fig. 4d–e). Additionally, assessment of brain cysts revealed a complete absence of cyst formation in immunized mice challenged with 5 × 10^4^ Pru strains, significantly decreased compared to that of the naive mice (Fig. 4f). In summary, vaccination with PruΔ*gra72* in mice can confer effective immune protection against *T. gondii* acute infection by tachyzoites of less virulent strains (PYS and Pru) and partial protection against infection caused by tachyzoites of the virulent RH strain.

### PruΔ*gra72* confers protection against chronic infection by cysts in mice

To further assess the potential application of PruΔ*gra72* in protection against chronic *T. gondii* infection, 10 or 40 Pru cysts were orally administered to the immunized mice and naive mice at 60 dpv (Fig. 3). All the immunized mice survived after challenge with low or high doses of cysts. However, the unvaccinated mice exhibited clinical symptoms at 8 dpi, with ruffled fur and reduced appetite, and began dying at 11 dpi, with survival rates of 40% and 30% at low and high doses, respectively (Fig. 4g–h). These findings indicate that immunization with PruΔ*gra72* conferred protective efficacy against chronic infection in mice.

### PruΔ*gra72* vaccination decreases brain cyst burden of chronically infected mice

The brain tissue of all surviving mice at 30 days in 60 dpv challenged groups were collected and used to determine the parasite cyst numbers. Non-immunized mice exposed to low-dose cysts showed an average cyst count of 346 ± 135 and 626 ± 215 cysts under high-dose cyst attack (Fig. 4i). However, the immunized mice demonstrated markedly reduced cyst burdens, with averages of merely 3 ± 8 and 5 ± 9 cysts in the immunized mice challenged with low and high dose of Pru cysts, respectively (Fig. 4i). Statistical analysis reveals a significant difference of brain cyst burden between the vaccinated and non-vaccinated groups (*P* < 0.0001). The positive rates of *B1* gene in brain tissue of all immunized mice challenged by 10 and 40 cysts were 60%, suggesting that although most immunized mice brains were infected with *T. gondii*, the parasites failed to form brain cysts (Additional file 4: Figure S3 and Additional file 5: Table S2). These results suggest that immunization with PruΔ*gra72* tachyzoites reduces the brain cyst burden of chronically *T. gondii*-infected mice.

### Immunization with PruΔ*gra72* provides long-term protection against *T. gondii* infection

**Fig. 5:**
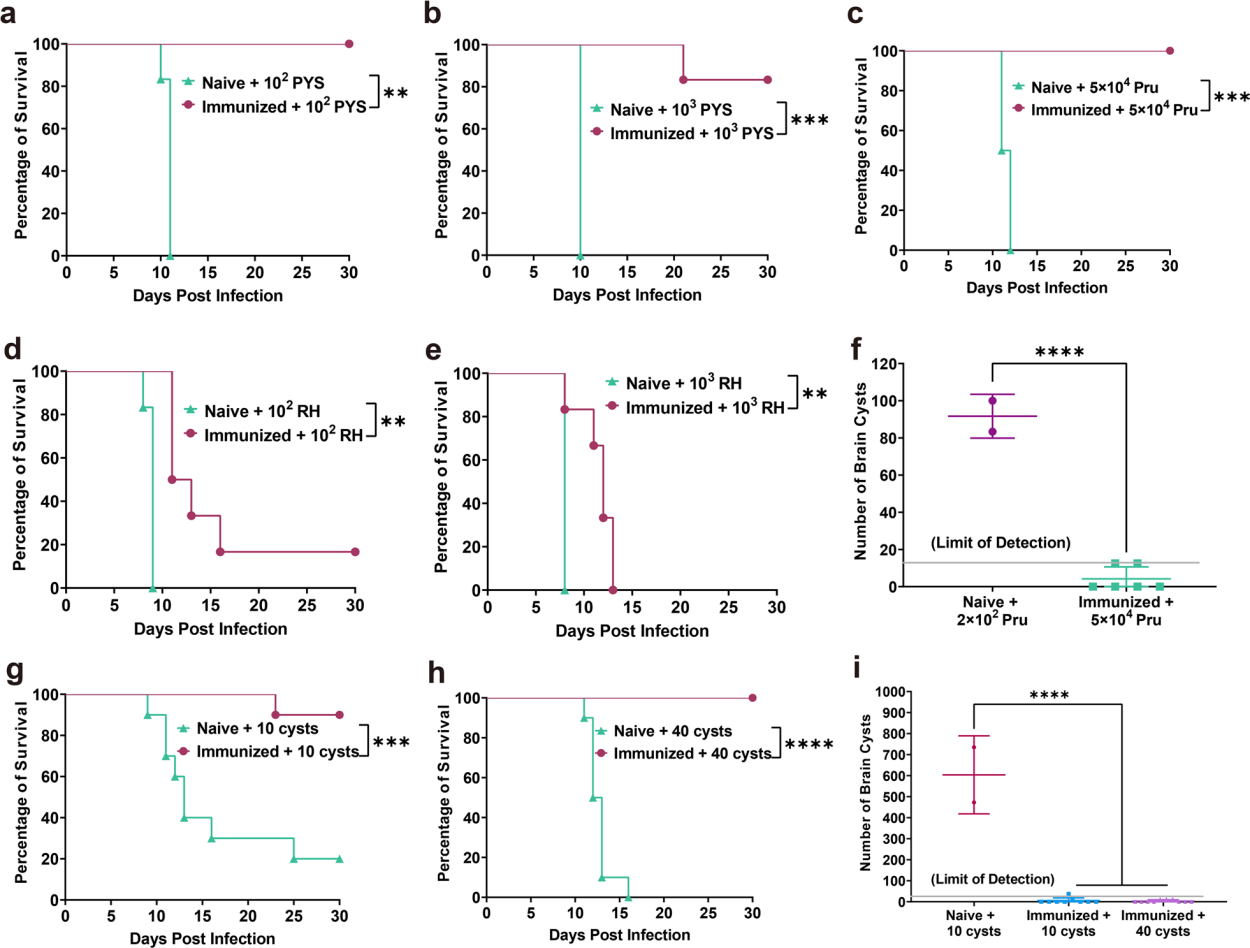
Immunization with PruΔ*gra72* induced enduring protection against *Toxoplasma gondii* infection by tachyzoites and cysts. **a–e** Mice were challenged with 10^2^ or 10^3^ tachyzoites of the ToxoDB#9 PYS strain, 5 × 10^4^ tachyzoites of the Type II Pru strain or 10^2^ or 10^3^ tachyzoites of the Type I RH strain at 120 days post-vaccination (dpv). Clinical symptoms and survival statuses were recorded for 30 days (*n* = 6 mice per group). ***P < 0.001, **P < 0.01. **f** Brain cyst numbers in mice infected with 5 × 10^4^ Pru tachyzoites at 30 days after infection and mice infected with 5 × 10^2^ Pru tachyzoites served as the control group. ****P < 0.0001. The survival rates of mice orally administered 10 cysts (**g**) or 40 cysts (**h**) were recorded over a 1-month period (*n* = 10 mice per group). ****P < 0.0001, ***P < 0.001. **i** Brain cyst burden was quantified at 30 days post-infection from both non-immunized and immunized and surviving mice. ****P < 0.0001

To further investigate whether vaccination with PruΔ*gra72* provides long-term protection against *T. gondii* infection in mice, three genotypes of *T. gondii* tachyzoites with different virulence (Type I RH strain, Type II Pru strain and ToxoDB#9 PYS strain) were intraperitoneally injected into Kunming mice at 120 dpv to observe their clinical symptoms and survival rates for 30 days (Fig. 3). While all non-immunized mice were killed when challenged with the same dose of tachyzoites of PYS or Pru strains (Fig. 5a–c), the immunized mice showed 100% survival rates when challenged with 10^2^ PYS or 5 × 10^4^ Pru tachyzoites (Fig. 5a, c). When challenged by 10^3^ tachyzoites of the PYS strain, only one mouse in the immunized group died on the 21st day, maintaining a high survival rate of 83.3% (Fig. 5b). However, PruΔ*gra72* vaccination did not effectively protect against challenge infection with the virulent RH strain, and the immunized mice challenged with 10^2^ tachyzoites of the RH strain displayed a survival rate of only 16.7%, and all mice challenged by 10^3^ tachyzoites of the RH strain succumbed to the infection (Fig. 5d–e). Despite the relatively low survival rates compared to that in mice groups challenged with PYS or Pru strains, PruΔ*gra72* vaccination still extended the survival time of mice challenged with the RH strain.

For assessment of long-term protection against *T. gondii* cyst infection, 10 or 40 cysts were used to orally infect the naive and immunized mice (Fig. 3). The immunized group displayed high survival rates of 90% and 100%, respectively, while the survival rates of the naive group challenged with 10 and 40 Pru cysts were 20% and 0% (Fig. 5g–h). The surviving naive mice had an average brain cyst burden of 604 ± 186, while the immunized group challenged with 10 and 40 cysts had brain cyst burdens of 6 ± 13 and 4 ± 6 per brain, respectively (Fig. 5i). The positivity rates of brain *B1* gene in these surviving mice were 66.7% and 80%, respectively (Additional file 4: Figure S3 and Additional file 5: Table S2), showing an increase compared to that at 60 dpv, which may possibly have resulted from the decreased resistance to cysts due to the prolonged immunization time. These results indicate that PruΔ*gra72* vaccination could not provide complete defense against infection with tachyzoites of the virulent RH strain but can offer long-term protection against low-virulent *T. gondii* tachyzoites and cyst-induced chronic infections.

### Vaccination stimulates a notable specific immune response

**Fig. 6:**
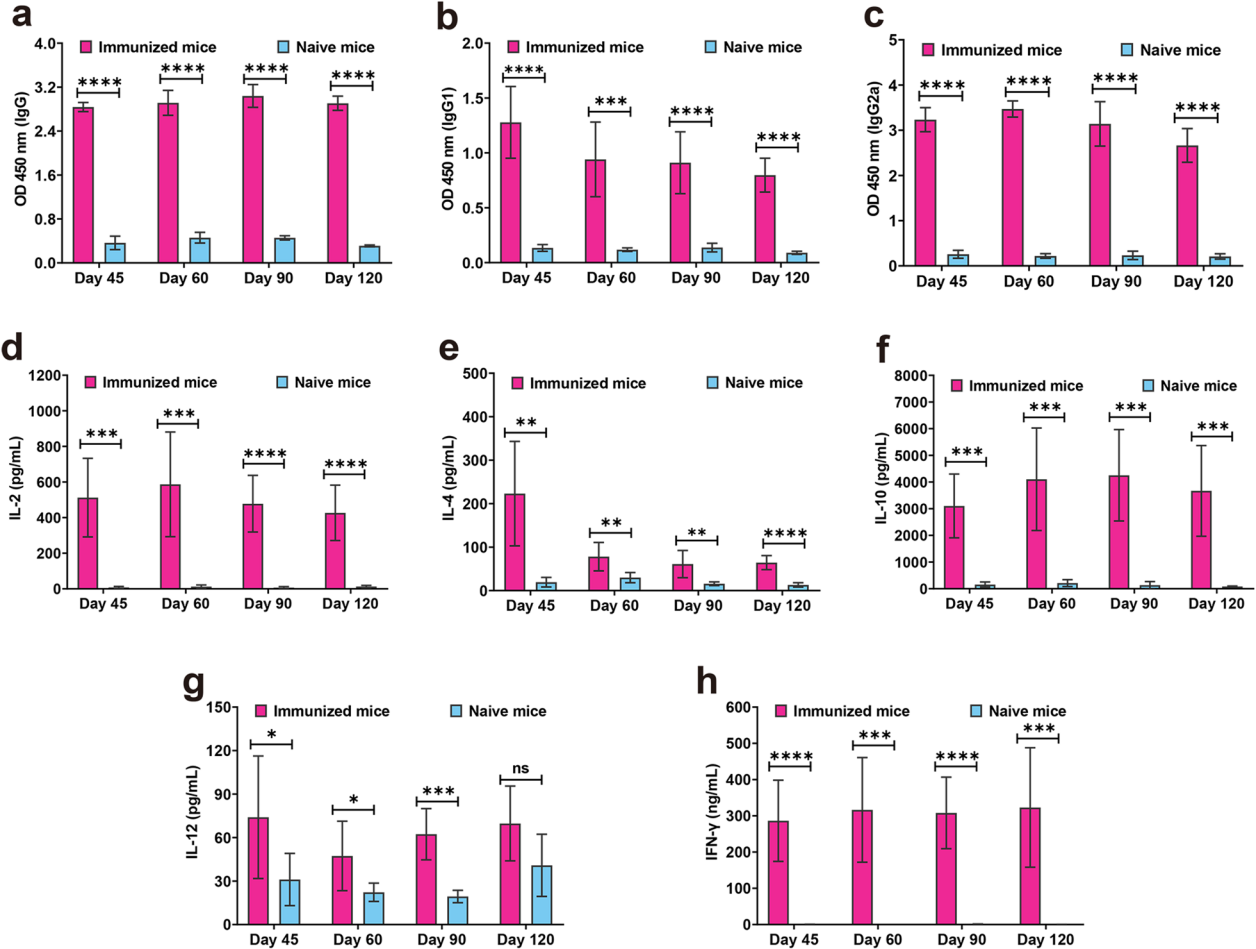
PruΔ*gra72* vaccination elicits Th1/Th2-type immune responses in mice. **a–c** The IgG **(a)**, IgG1 **(b)** and IgG2a **(c)** levels of the sera from the immunized and naive mice collected at 45, 60, 90 and 120 days post-vaccination (dpv) (*n* = 6 mice per group). Mice immunized with PruΔ*gra72* displayed significantly elevated levels of IgG and IgG subclasses compared to the control group. ****P < 0.0001, ***P < 0.001. **d–h** The Th1 and Th2 cytokine levels of the splenocytes from the immunized and naive mice collected at 45, 60, 90 and 120 dpv, including IL-2 (**d**), IL-4 (**e**), IL-10 (**f**) and IL-12 (**g**) and IFN-γ (**h**) (n = 6 mice per group). ****P < 0.0001, ***P < 0.001, **P < 0.01, *P < 0.05. *ns* indicates no statistical significance

To characterize the immunogenicity of PruΔ*gra72* vaccination, serum samples were collected at 45, 60, 90 and 120 dpv to monitor levels of *T. gondii*-specific IgG and IgG subclasses by quantitative ELISA (Fig. 3). The results showed that PruΔ*gra72* vaccination induced significantly high levels of anti-*T. gondii*-specific IgG and IgG subclasses (IgG1 and IgG2a) in mice at all time points compared to that of the naive mice (Fig. 6a–c). Furthermore, IgG2a level was significantly higher than IgG1 level in all vaccination time points (Additional file 6: Figure S4a). These results demonstrated that PruΔ*gra72* vaccination induces balanced Th1 and Th2 responses, with a predilection towards Th1 dominance.

To further elucidate the immune response, splenocytes of the immunized and naive mice collected at 45, 60, 90 or 120 dpv were cultured *in vitro* and stimulated with STAg to detect cytokines by ELISA (Fig. 3). Th1-type cytokines (IL-2, IL-12 and IFN-γ) and Th2-type cytokines (IL-4 and IL-10) were significantly upregulated by PruΔ*gra72* vaccination at all time points, except IL-12 level at 120 dpv (Fig. 6d–h). The level of IFN-γ was the most prominent among all examined cytokines, significantly surpassing that of other cytokines such as IL-2, IL-4, IL-10 and IL-12 (Additional file 6: Figure S4b–e).

## Discussion

In recent years, numerous GRAs have been proven as promising candidate proteins for developing *T. gondii* vaccines. Among them, GRA7 can be used to develop a DNA vaccine to trigger a strong antibody response and higher levels of IFN-γ [36]. The recombinant protein GRA12 enclosed in PLGA nanoparticles shows effectiveness as a vaccine against acute toxoplasmosis [37]. The live-attenuated RHΔ*gra17* induces high levels of Th1 and Th2 cell-mediated immune responses and provides some degree of protection against homologous and heterologous virulent strains in mice [38].

Previous studies have indicated the significance of the novel GRA72 in modulating the permeability of the PVM and its association with the growth of *T. gondii* [26, 27]. Gene knockout strains like PruΔ*gra72* or ME49Δ*gra72* exhibit reduced virulence in mice, suggesting their potential as live-attenuated vaccines. In this study, virulence assays showed that Kunming mice survived after infection with even 5 × 10^7^ PruΔ*gra72* tachyzoites, with slight clinical symptoms of toxoplasmosis, but no brain cysts were observed. Additionally, deletion of the *gra72* gene in the Pru strain significantly reduced parasite load in organs of the infected mice. The PruΔ*gra72* strain has the potential as a toxoplasmosis vaccine candidate.

Studies of genetic diversity of *T. gondii* isolates have revealed the existence of around 300 different genotypes; some of them are quite different in their virulence to mice [4–6, 39]. In our study, we found that PruΔ*gra72* vaccination fully protected mice against attacks by Type II Pru strain and ToxoDB#9 PYS strain and prolonged the survival time of mice infected by Type I RH strain. These results indicated that PruΔ*gra72* can protect mice against acute infection by low-virulent *T. gondii* strains. Currently, less virulent candidate vaccine strains such as ME49Δ*cdpk3*, PruΔ*gra76* and RHΔ*tkl1* have been evaluated for their protective efficacy against tachyzoite infection using mouse models [16, 17, 40]. Among them, ME49Δ*cdpk3* and PruΔ*gra76* did not provide 100% protection against the highly virulent RH strain, similar to PruΔ*gra72* [17, 40]. However, RHΔ*tkl1* offers sufficient protection for mice against Type I RH or ToxODB#9 (PYS or TGC7) tachyzoites, in which all RHΔ*tkl1*-immunized mice survived the challenge infection of tachyzoites of the highly virulent Type I RH strain [16]. Immunization with *T. gondii* uracil auxotrophs, which were completely avirulent because of the lack of carbamoyl phosphate synthetase II (CPSII) gene, induces long-term protective immunity against RH strain in mice [41].

The persistent presence of cysts containing *T. gondii* bradyzoites causes chronic infection, posing substantial health risks to the host [42]. We simulated the chronic infection in mice by orally administering Pru cysts at 60 or 120 dpv. The results showed that PruΔ*gra72* vaccination significantly improved the survival rates and decreased the brain cyst burden of mice chronically infected by Pru cysts. The effective protection against chronic *T. gondii* infection might be associated with the strong humoral and cellular immune responses elicited in mice by the vaccination, in which the Th1 response has been shown to be beneficial in both the clearance of tachyzoites during the acute phase and the suppression of cyst formation [43, 44]. Consequently, vaccination with PruΔ*gra72* can effectively combat low-virulent strains and chronic infections. Nonetheless, it is imperative to acknowledge the study’s limitations, notably the absence of assessment regarding congenital infections and chronic infection by oocysts, two important *T. gondii* infection routes, which warrants further investigation.

Immune response skewed towards Th1 has advantages in enhancing the host defense against *T. gondii* [43, 44]. Consistent with results of previous studies of live-attenuated vaccines, such as ME49Δ*cdpk3*, ME49Δ*α-amy* and RHΔ*ompdc*Δ*uprt* [17, 33, 35], mice administered PruΔ*gra72* displayed sustained and elevated levels of IgG and IgG subclass antibodies at 45, 60, 90 and 120 dpv. Despite a marked increase in both IgG1 and IgG2a levels in immunized mice compared to that of the non-immunized mice, IgG2a levels were significantly higher than IgG1 levels, suggesting a prevailing Th1 response among the coexistence of Th1 and Th2 immune reactions in mice vaccinated with PruΔ*gra72*, consistent with previous findings [35, 38]. These antibodies may proficiently obstruct the host cell invasion by the parasites, opsonize the parasites through phagocytosis mechanisms and activate the classical complement pathway to effectively defend against *T. gondii* [8].

IL-12 and IFN-γ exert pivotal roles in driving Th1 cell-mediated immunity and are essential cytokines for controlling parasitic infections [42, 45]. Mice lacking the IFN-γ gene or receptor are vulnerable to death after *T. gondii* infection [42]. IL-2 is an additional key factor in the protective response [8]. In this study, the levels of IFN-γ and IL-2 detected in the splenocyte supernatants were significantly increased by PruΔ*gra72* vaccination. The increased expression level of cytokines might play important roles in immune protection, enabling the mice to resist *T. gondii* infections. Previous studies have shown a significant correlation between IFN-γ and IgG2a antibody production [46]. This is consistent with our results as the level of IgG2a is significantly higher than that of IgG1. Similarly, the level of IFN-γ is also significantly higher than that of other cytokines. In mice, both cellular and humoral immune responses tend to skew towards a Th1-type response.

Excessive immune responses pose a potential threat; therefore, regulating host-specific immunity is crucial for the overall survival of the host [47]. IL-10 plays a key role in alleviating tissue damage caused by strong Th1-type responses, inhibiting lymphocytes from secreting IFN-γ, while IL-4 also plays a similar role [42]. Our study found that the levels of Th2-type anti-inflammatory cytokines (IL-4 and IL-10) were elevated after PruΔ*gra72* vaccination, which may alleviate the increase of pro-inflammatory mediators and reduce collateral damage to the mice and help to better regulate Th1-type cytokines.

In addition to live attenuated *T. gondii* vaccines, DNA vaccines and protein vaccines have also been investigated in animal models. Consistent with immune responses induced by live attenuated vaccines, several DNA or protein vaccines also induce Th1-type cellular and humoral immune response, such as the DNA vaccine expressing heat shock protein 40 (HSP40) [48], a double C2 domain protein (DOC2) or rhoptry protein 9 (ROP9) [49, 50], protein vaccine of recombinant extracellular signal-regulated kinases 7 (ERK7) or calcium-dependent protein kinase 1 (CDPK1) [51, 52]. However, protection against *T. gondii* induced by these DNA or protein vaccines was not 100% because of incomplete protective immunity induced by single or multiple genes/proteins. In the present study, although significant protection against *T. gondii* in mice was induced by PruΔ*gra72* vaccination, this live attenuated PruΔ*gra72* vaccine may be practically useful for food-producing animals and cats, but not for humans, because of its resistance to pyrimethamine.

## Conclusions

The findings of our study showed that PruΔ*gra72* vaccination triggers the host immune responses, eliciting a balanced Th1/Th2 immune reaction that confers long-lasting immunity against both mild and relatively virulent *T. gondii* strains, resulting in the prolonged survival time, improved survival rates and decreased brain cyst burden. As such, PruΔ*gra72* represents a promising candidate as a potentially live-attenuated vaccine. Nevertheless, the mouse model in this study showcases the immunogenicity and effectiveness of PruΔ*gra72*; further assessment in diverse susceptible and economically important animal models is imperative to ascertain its safety and efficacy. Furthermore, despite the effective protection against chronic infection displayed by PruΔ*gra72* immunization, the persistence of brain cysts remains a concern. Low level protection against infection by tachyzoites of the highly virulent RH strain warrants continued research to advance the development of live-attenuated vaccines against *T. gondii*.

## Supplementary Information

Additional file 1: Figure S1. Generation of the *gra72* knockout strain in Type II Pru strain of *Toxoplasma* *gondii*. (a) Schematic illustration of constructing the mutant strain using CRISPR/Cas9-mediated homologous recombination to disrupt the *gra72* gene and replace the conding sequence with a DHFR cassette conferring resistance to pyrimethamine. (b) PCR identification of PruΔ*gra72* knockout strain. PCR1 and PCR3 were utilized to discern the integration of 5′ and 3′ homologous DHFR cassette targeting the *gra72* gene, while PCR2 was employed to validate the successful knockout of *gra72*. (c) Morphological characterization of the PVs fromed by PruΔ*gra72* and Pru tachyzoites in HFF cells at 60 h post-infection. In comparison to the wild-type Pru strain, the PruΔ*gra72* exhibited a “bubble” morphology of PVs. Scale bars, 10 µm.

Additional file 2: Figure S2. The results of PCR targeting *B1* gene in brain tissues of mice infected with 2 × 10^2^ (a), 5 × 10^2^ (a), 5 × 10^3^ (b), 5 × 10^4^ (b), 5 × 10^5^ (c), 5 × 10^6^ (c) and 5 × 10^7^ (d) PruΔ*gra72* tachyzoites, showing an increasing positivey rate for *Toxoplasma* *gondii* infection with higher infective doses. A1–8, results from the surviving mice infected with 2 × 10^2^ PruΔ*gra72* tachyzoites; B1–8, results from the surviving mice infected with 5 × 10^2^ PruΔ*gra72* tachyzoites; C1–8, results from the surviving mice infected with 5 × 10^3^ PruΔ*gra72* tachyzoites; D1–8, results from the surviving mice infected with 5 × 10^4^ PruΔ*gra72* tachyzoites; E1–8, results from the surviving mice infected with 5 × 10^5^ PruΔ*gra72* tachyzoites; F1–8, results from the surviving mice infected with 5 × 10^6^ PruΔ*gra72* tachyzoites; G18, results from the surviving mice– infected with 5 × 10^7^ PruΔ*gra72* tachyzoites. P, positive control; N, negative control.

Additional file 3: Table S1. Brain cyst burden and B1 gene detection results in mice infected with Pru or PruΔ*gra72* tachyzoites in virulence assays.

Additional file 4: Figure S3. The results of PCR targeting *B1* gene in brain tissues of mice immunized with PruΔ*gra72* and challenged with Pru tachyzoites (a) and cysts (b–c). A1–6, results from the surviving mice challenged with 5 × 10^4^ Pru tachyzoites at 60 days post-vaccination (dpv); B1–6, results from the surviving mice challenged with 5 × 10^4^ Pru tachyzoites at 120 dpv; C1–10, results from the surviving mice challenged with 10 cysts at 60 dpv; D1–10, results from the surviving mice challenged with 40 cysts at 60 dpv; E1–9, results from the surviving mice challenged with 10 cysts at 120 dpv; F1–10, results from the surviving mice challenged with 40 cysts at 120 dpv. P, positive control; N, negative control.

Additional file 5: Table S2. Brain cyst burden and B1 gene detection results in mice immunized with PruΔ*gra72* and challenged with Pru tachyzoites or cysts.

Additional file 6: Figure S4. Levels of IgG2a antibodies in the PruΔ*gra72*-immunized mouse group were significantly higher than levels of IgG1 antibodies at 45, 60, 90 and 120 days post-vaccination (dpv) (a). ****P < 0.0001. Level of IFN-γ significantly surpasses that of other cytokines (IL-2, IL-4, IL-10 and IL-12) at 45, 60, 90 and 120 dpv (b–e). ****P < 0.0001, ***P < 0.001.

## Data Availability

The datasets supporting the findings of this article are included within the paper and its supplementary materials.

## Abbreviations

PV: Parasitophorous vacuole
IgG: Immunoglobulin G
IL-2: Interleukin-2
IL-12: Interleukin-12
IFN-γ: Interferon-γ
IL-4: Interleukin-4
IL-10: Interleukin-10
GRAs: Dense granule proteins
IVN: Intravacuolar network
PVM: Parasitophorous vacuole membrane
HFF: Human foreskin fibroblast
DMEM: Dulbecco’s Modified Eagle Medium
FBS: Fetal bovine serum
qPCR: Quantitative polymerase chain reaction
dpi: Days post-infection
dpv: Days post-vaccination
STAg: Soluble *T. gondii* antigen
ELISA: Enzyme-linked immunosorbent assay
PBST: Phosphate buffered saline with tween
BSA: Bovine serum albumin
TMB: 3, 3′, 5, 5′-Tetramethylbenzidine
OD: Optical density
