# Supplementary figures and images for: Live-attenuated PruΔgra72 strain of Toxoplasma gondii induces strong protective immunity against acute and chronic toxoplasmosis in mice
Source: Parasit Vectors. 2024 Sep 5;17:377. doi: 10.1186/s13071-024-06461-9 (PMC11378421; doi:10.1186/s13071-024-06461-9)

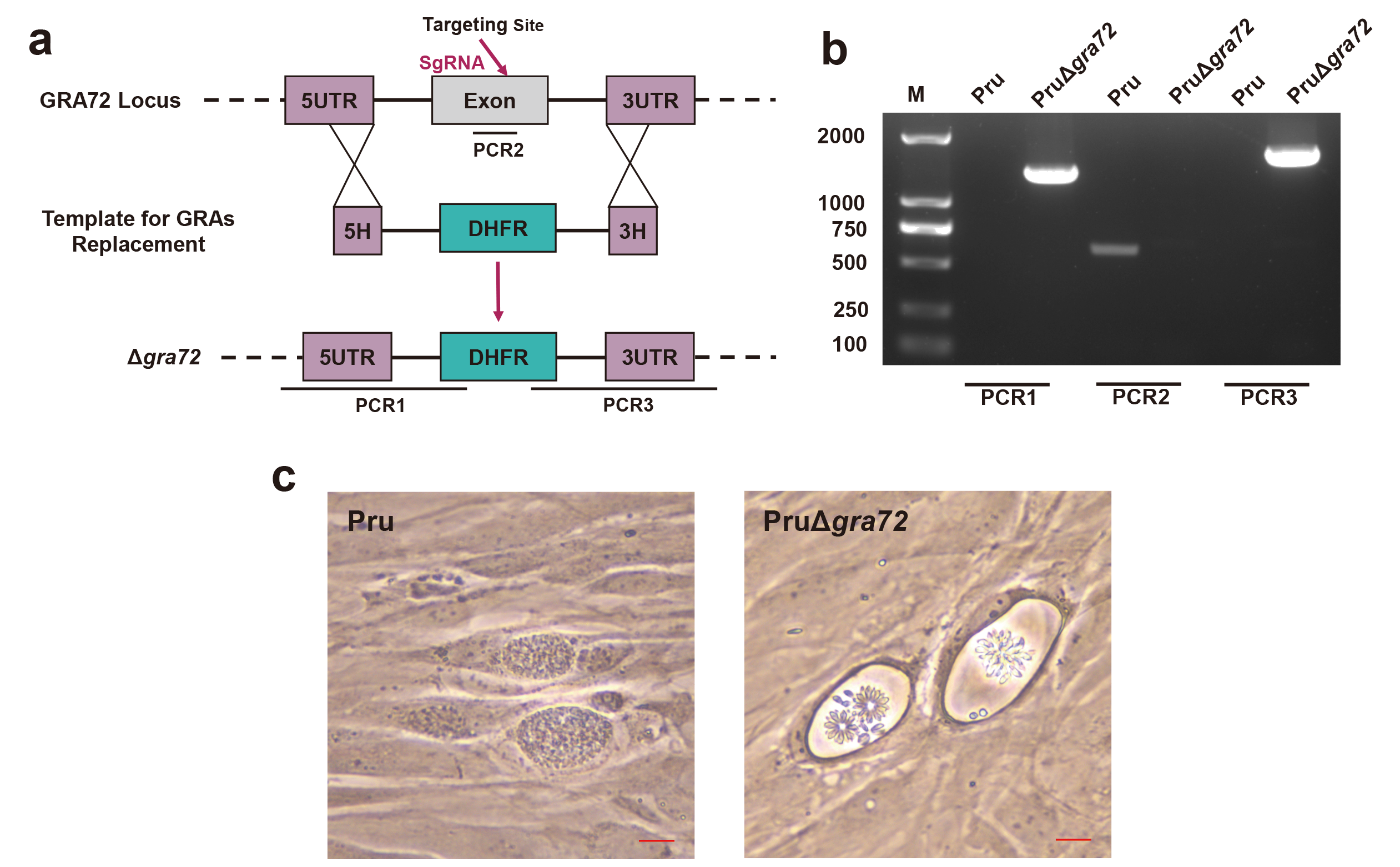

Supplement: Supplementary file 1 — Additional file 1: Figure S1. Generation of the gra72 knockout strain in Type II Pru strain of Toxoplasma gondii. (a) Schematic illustration of constructing the mutant strain using CRISPR/Cas9-mediated homologous recombination to disrupt the gra72 gene and replace the conding sequence with a DHFR cassette conferring resistance to pyrimethamine. (b) PCR identification of PruΔgra72 knockout strain. PCR1 and PCR3 were utilized to discern the integration of 5′ and 3′ homologous DHFR cassette targeting the gra72 gene, while PCR2 was employed to validate the successful knockout of gra72. (c) Morphological characterization of the PVs fromed by PruΔgra72 and Pru tachyzoites in HFF cells at 60 h post-infection. In comparison to the wild-type Pru strain, the PruΔgra72 exhibited a “bubble” morphology of PVs. Scale bars, 10 µm. [file 13071_2024_6461_MOESM1_ESM.tif]

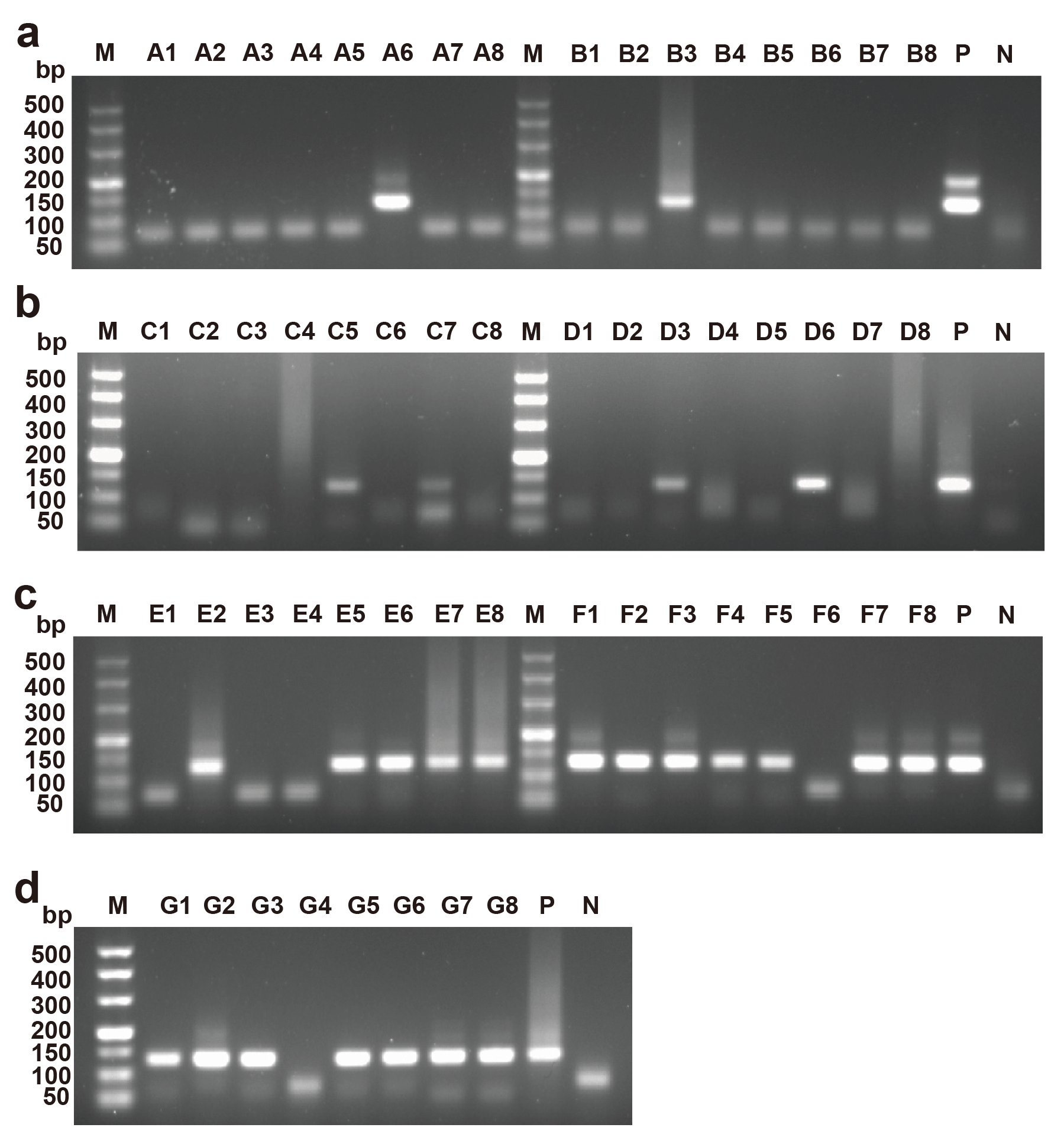

Supplement: Supplementary file 2 — Additional file 2: Figure S2. The results of PCR targeting B1 gene in brain tissues of mice infected with 2 × 10^2^ (a), 5 × 10^2^ (a), 5 × 10^3^ (b), 5 × 10^4^ (b), 5 × 10^5^ (c), 5 × 10^6^ (c) and 5 × 10^7^ (d) PruΔgra72 tachyzoites, showing an increasing positivey rate for Toxoplasma gondii infection with higher infective doses. A1–8, results from the surviving mice infected with 2 × 10^2^ PruΔgra72 tachyzoites; B1–8, results from the surviving mice infected with 5 × 10^2^ PruΔgra72 tachyzoites; C1–8, results from the surviving mice infected with 5 × 10^3^ PruΔgra72 tachyzoites; D1–8, results from the surviving mice infected with 5 × 10^4^ PruΔgra72 tachyzoites; E1–8, results from the surviving mice infected with 5 × 10^5^ PruΔgra72 tachyzoites; F1–8, results from the surviving mice infected with 5 × 10^6^ PruΔgra72 tachyzoites; G18, results from the surviving mice– infected with 5 × 10^7^ PruΔgra72 tachyzoites. P, positive control; N, negative control. [file 13071_2024_6461_MOESM2_ESM.tif]

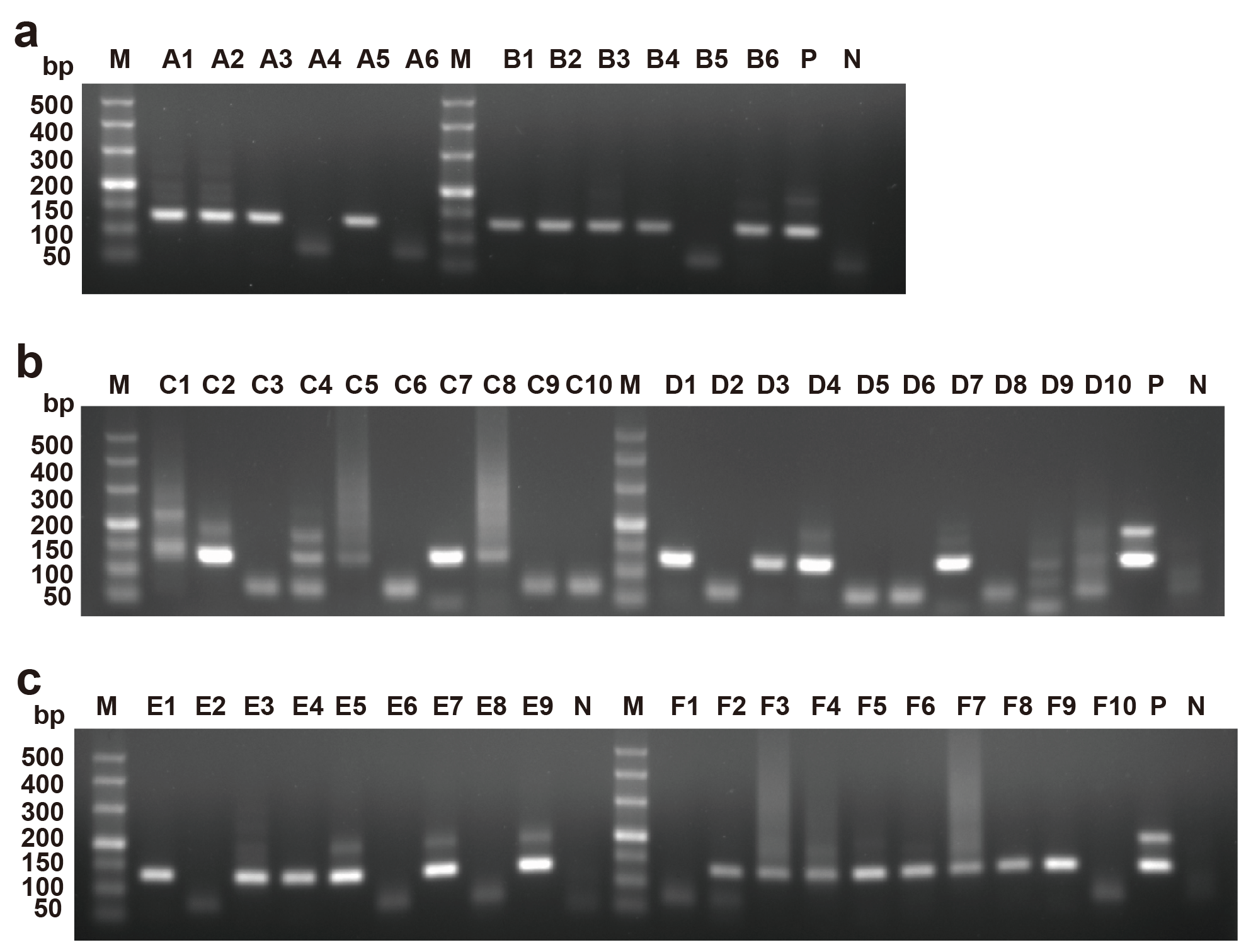

Supplement: Supplementary file 4 — Additional file 4: Figure S3. The results of PCR targeting B1 gene in brain tissues of mice immunized with PruΔgra72 and challenged with Pru tachyzoites (a) and cysts (b–c). A1–6, results from the surviving mice challenged with 5 × 10^4^ Pru tachyzoites at 60 days post-vaccination (dpv); B1–6, results from the surviving mice challenged with 5 × 10^4^ Pru tachyzoites at 120 dpv; C1–10, results from the surviving mice challenged with 10 cysts at 60 dpv; D1–10, results from the surviving mice challenged with 40 cysts at 60 dpv; E1–9, results from the surviving mice challenged with 10 cysts at 120 dpv; F1–10, results from the surviving mice challenged with 40 cysts at 120 dpv. P, positive control; N, negative control. [file 13071_2024_6461_MOESM4_ESM.tif]

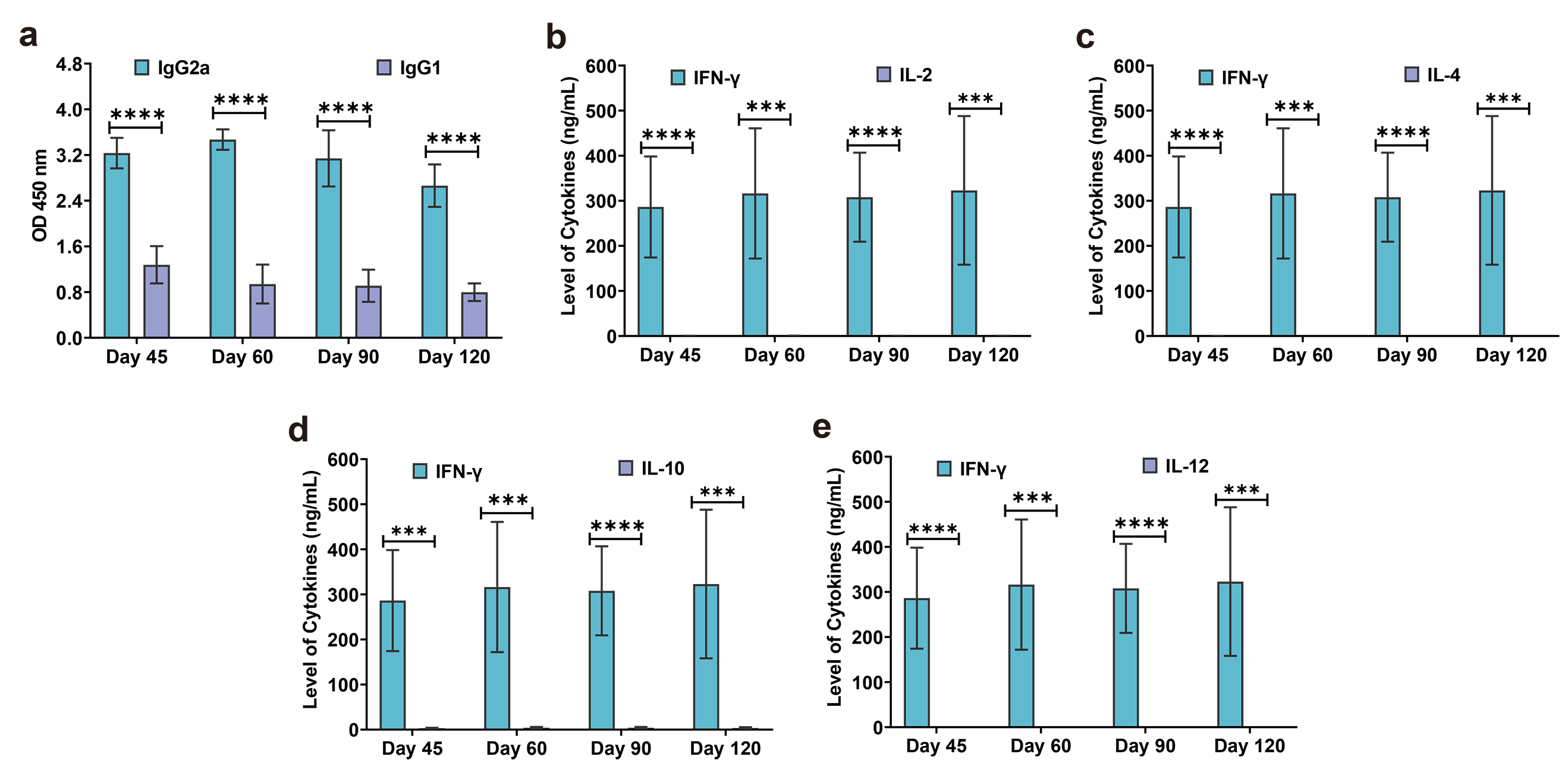

Supplement: Supplementary file 6 — Additional file 6: Figure S4. Levels of IgG2a antibodies in the PruΔgra72-immunized mouse group were significantly higher than levels of IgG1 antibodies at 45, 60, 90 and 120 days post-vaccination (dpv) (a). ****P < 0.0001. Level of IFN-γ significantly surpasses that of other cytokines (IL-2, IL-4, IL-10 and IL-12) at 45, 60, 90 and 120 dpv (b–e). ****P < 0.0001, ***P < 0.001. [file 13071_2024_6461_MOESM6_ESM.tif]
