# Supplementary material for: Live-attenuated PruΔgra72 strain of Toxoplasma gondii induces strong protective immunity against acute and chronic toxoplasmosis in mice
Source: Parasit Vectors. 2024 Sep 5;17:377. doi: 10.1186/s13071-024-06461-9 (PMC11378421; doi:10.1186/s13071-024-06461-9)
Supplement: Supplementary file 3 — Additional file 3: Table S1. Brain cyst burden and B1 gene detection results in mice infected with Pru or PruΔgra72 tachyzoites in virulence assays. [file 13071_2024_6461_MOESM3_ESM.pdf]

Additional file 3: Table S1. Brain cyst burden and *B1* gene detection results in mice infected with Pru or PruΔ*gra72* tachyzoites in virulence assays

Group	Average brain cysts number	Positive rate of *B1* gene (%)	Brain cyst number (*B1* gene detection result)							
			Mouse 1	Mouse 2	Mouse 3	Mouse 4	Mouse 5	Mouse 6	Mouse 7	Mouse 8
Pru	92	100	83	100	-	-	-	-	-	-
2×10^2 PruΔ*gra72*	0	12.5	0 (-)	0 (-)	0 (-)	0 (-)	0 (-)	0 (+)	0 (-)	0 (-)
5×10^2 PruΔ*gra72*	0	12.5	0 (-)	0 (-)	0 (+)	0 (-)	0 (-)	0 (-)	0 (-)	0 (-)
5×10^3 PruΔ*gra72*	0	25.0	0 (-)	0 (-)	0 (-)	0 (-)	0 (+)	0 (-)	0 (+)	0 (-)
5×10^4 PruΔ*gra72*	0	25.0	0 (-)	0 (-)	0 (+)	0 (-)	0 (-)	0 (+)	0 (-)	0 (-)
5×10^5 PruΔ*gra72*	0	62.5	0 (-)	0 (+)	0 (-)	0 (-)	0 (+)	0 (+)	0 (+)	0 (+)
5×10^6 PruΔ*gra72*	0	87.5	0 (+)	0 (+)	0 (+)	0 (+)	0 (+)	0 (-)	0 (+)	0 (+)
5×10^7 PruΔ*gra72*	0	87.5	0 (+)	0 (+)	0 (+)	0 (-)	0 (+)	0 (+)	0 (+)	0 (+)
