# Supplementary material for: Live-attenuated PruΔgra72 strain of Toxoplasma gondii induces strong protective immunity against acute and chronic toxoplasmosis in mice
Source: Parasit Vectors. 2024 Sep 5;17:377. doi: 10.1186/s13071-024-06461-9 (PMC11378421; doi:10.1186/s13071-024-06461-9)
Supplement: Supplementary file 5 — Additional file 5: Table S2. Brain cyst burden and B1 gene detection results in mice immunized with PruΔgra72 and challenged with Pru tachyzoites or cysts. [file 13071_2024_6461_MOESM5_ESM.pdf]

Additional file 5: Table S2. Brain cyst burden and *B1* gene detection results in mice immunized with PruΔ*gra72* and challenged with Pru tachyzoites or cysts

**Group**	**Average brain cyst number**	**Positive rate of *B1* gene (%)**	**Brain cyst number (*B1* gene detection result)**									
			**Mouse 1**	**Mouse 2**	**Mouse 3**	**Mouse 4**	**Mouse 5**	**Mouse 6**	**Mouse 7**	**Mouse 8**	**Mouse 9**	**Mouse 10**
Immunized + Pru tachyzoites (60 d)	0	66.7	0 (+)	0 (+)	0 (+)	0 (-)	0 (+)	0 (-)				
Immunized + Pru tachyzoites (120 d)	0	83.3	0 (+)	0 (+)	0 (+)	0 (+)	0 (-)	0 (+)				
Naive +10 cysts (60 d)	346	100	500	288	250	-	-	-	-	-	-	-
Immunized +10 cysts (60 d)	3	60.0	0 (+)	25	0 (-)	0 (+)	0 (+)	0 (-)	0 (+)	0 (+)	0 (-)	0 (-)
Naive +40 cysts (60 d)	626	100	469	796	413	825	-	-	-	-	-	-
Immunized + 40 cysts (60 d)	5	60.0	19	0 (-)	15	19	0 (-)	0 (-)	0 (+)	0 (-)	0 (+)	0 (+)
Naive +10 cysts (120 d)	604	100	735	473	-	-	-	-	-	-	-	-
Immunized +10 cysts (120 d)	6	66.7	38	0 (-)	14	0 (+)	0 (+)	0 (-)	0 (+)	0 (-)	0 (+)	-
Immunized + 40 cysts (120 d)	4	80.0	0 (-)	0 (+)	0 (+)	13	0 (+)	0 (+)	0 (+)	13	13	0 (-)
